# Supplementary material for: Entangled N-photon states for fair and optimal social decision making
Source: Sci Rep. 2020 Nov 24;10:20420. doi: 10.1038/s41598-020-77340-3 (PMC7686359; doi:10.1038/s41598-020-77340-3)
Supplement: Supplementary file 1 — Supplementary information. [file 41598_2020_77340_MOESM1_ESM.docx]

**Supplementary information:**

**Entangled *N*-photon states for fair and optimal social decision making**

**Nicolas Chauvet^1*^,** **Guillaume Bachelier­­­^2^, Serge Huant^2^, Hayato Saigo^3^, Hirokazu Hori^4^, Makoto Naruse^1^**

*^1^* *Department of Information Physics and Computing, Graduate School of Information Science and Technology, The University of Tokyo, 7-3-1 Hongo, Bunkyo-ku, Tokyo 113-8656, Japan*

*^2^ Univ. Grenoble Alpes, CNRS, Institut Néel, 38000 Grenoble, France*

*^3^ Nagahama Institute of Bio-Science and Technology, 1266 Tamura, Nagahama, Shiga 526-0829, Japan*

*^4^ Interdisciplinary Graduate School, University of Yamanashi, Takeda, Kofu, Yamanashi 400-8510, Japan*

*** [nicolas_chauvet@ipc.i.u-tokyo.ac.jp](mailto:nicolas_chauvet@ipc.i.u-tokyo.ac.jp)

**Section 1. Realignment algorithm**

This section contains the realignment algorithm to be used by each user, for any number of users *N*, written in pseudo-code. It has been implemented using Matlab version 2019a in this study. Tunable parameters for this code are N, registerSize, threshold and dTheta, respectively the number of users, the number of past successful rewards in memory, the minimum number of conflict events in memory before changing the angle, and the angle step. Parameter values used in our studies are given in the main text.

if lastReward != LOSS

conflict $=\left\{ \begin{aligned} 1 \mathrm{if} lastReward==WIN/N \\ 0 &\mathrm{otherwise} \end{aligned} \right.$

register = append(register,conflict); %Add last conflict to the register

register = register(max(1,end-registerSize):end); %Limit to registerSize

end if

if sum(register) >= threshold

newAngle = randomElement([0:dTheta:360]);

register = [];

end if

**Section 2. Realignment: N=4 case**

In this section, the realignment algorithm presented in section 1 is applied to the *N* = 4 case, for either $|A\rangle_{4}$ state defined by (26) or $|S\rangle_{4}$ state defined by (25). The goal is to estimate the efficiency of the algorithm and the requirement in terms of number of required cooperative users for the algorithm to converge to an optimal situation. For the comparison to be as reliable as possible, a set of 100 random initial 4-angle combinations is used for every configuration, with 20 different trials for each combination to average and estimate uncertainties over.

Figure S1 shows the averaged reward of each user from the angle combination obtained after trial *t* using input state $|A\rangle_{4}$ and , with logarithmic scale for the number of trials. In each sub-figure, the number of users who apply the realignment algorithm is changed, from one in (a) to four in (d), and in every case the averaged reward is estimated among the group of moving or fixed users by taking the angle combination at trial *t* and checking how much reward is obtained with this one after 1000 tries.

**Figure S1.** Individual reward averaged over groups of users who use the realignment algorithm (Moving user(s), blue) or not (Fixed user(s), red) on the state $|A\rangle_{4}$ with , for (a) one moving user, (b) two moving users, (c) three moving users and (d) four moving users.

As the main text describes, fairness is always reached within margins of error, which means that both fixed and moving users get the same average outcome. Besides, even when a single user applies the algorithm, optimal situation can be reached whatever the initial angle combination, with little to no difference in terms of limit and speed of convergence between one, two, three or four active users. This is expected from Fig. 7(a) of the main text, as any initial 4-angle combination is linked to an optimal plane by translation along, x, y or z axis, and similarly for user 4 due to the global rotation invariance.

**Figure S2.** Individual reward averaged over groups of users who use the realignment algorithm (Moving user(s), blue) or not (Fixed user(s), red) on the state $|A\rangle_{4}$ with , for (a) one moving user, (b) two moving users, (c) three moving users and (d) four moving users.

The same simulations have been run with the state $|A\rangle_{4}$, this time with in (26) of the main text. Figures 7 already showed a major difference, that is the absence of plane of optimal configurations, replaced by lines in this 3-dimensional representation. As a consequence, any initial 4-angle combination cannot (in general) be linked to an optimal situation by only a 1-dimensional translation, this time needing 2-dimensional translation. Figures S2 illustrate this difference, as the situation for one moving user in (a) show a limit of convergence that is sub-optimal (250 reward per user in the optimal case, 242 on average here). Moreover, even with two or more moving users, the convergence is significantly slower than with the state at , optimal state being only reached after 10000 trials for here, whereas it is reached after about 1000 trials for . In both cases, these results indicate that it is in each user’s interest to apply the random correction algorithm, both individually and collectively, while not all users are required to cooperate for convergence to be obtained.

**
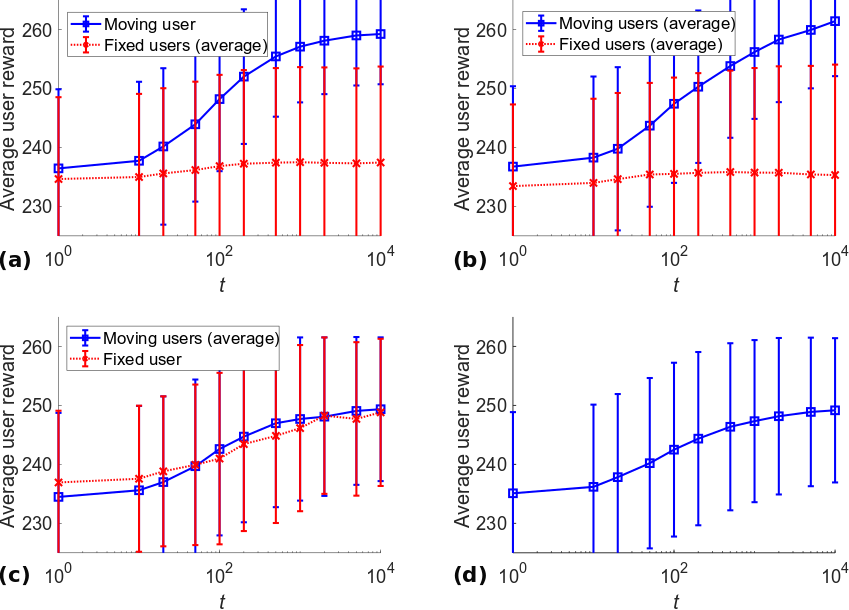
**

**Figure S3.** Individual reward averaged over groups of users who use the realignment algorithm (Moving user(s)) or not (Fixed user(s)) on the state $|S\rangle_{4}$, for (a) one moving user, (b) two moving users, (c) three moving users and (d) four moving users.

The situation is different with the state $|S\rangle_{4}$, as Figs. S3 illustrate. This time, fairness is not obtained in general, as can also be seen by the wider uncertainties on the individual user reward. Besides, if only one or two users move(s) while the others stay fixed, they can expect higher average reward than the optimal fair situation. When three or more users apply the algorithm however, convergence is observed, although fairness is not always observed. More importantly in this case, it is in the best interest of each user to try to persuade other users that they should stay fixed while they try to get a most favorable situation for themselves… In other words, the Nash equilibrium is not the best individual scenario anymore. Hence, state $|S\rangle_{4}$ should be avoided if the objective is to guarantee fairest repartition for all users.

**Section 3. State for N=5 case**

With 5 users, there are 4 kinds of state that can be involved instead of 3 for 3 users: $|HHHHV\rangle$, $|HHHVV\rangle$, $|HHVVV\rangle$ and $|HVVVV\rangle$, in addition to all their different permutations. Initially, we tried to find states with only $|HHHHV\rangle$/$|HVVVV\rangle$, or only $|HHHVV\rangle$/$|HHVVV\rangle$, but global rotation invariance makes it impossible. Thus, we have to find a state from all possible combinations of them and their permutations, with similar conditions as for cases *N*=3 and *N*=4.

From the previous cases, we hypothesized that the N^th^ complex roots of 1 would be involved and tried to find a possible state with proper phase combinations from $z=e^{i\frac{2\pi}{5}}$. By fixing $a_{1}=1/\sqrt{30}$ and following the same derivation of equations as previous cases, we find the state:

$$\begin{matrix} \sqrt{30}|\psi_{5}\rangle=|HHHHV\rangle+z|HHHVH\rangle+z^{2}|HHVHH\rangle+z^{3}|HVHHH\rangle+z^{4}|VHHHH\rangle\\ \pm i\left(|VVVVH\rangle+z|VVVHV\rangle+z^{2}|VVHVV\rangle+z^{3}|VHVVV\rangle+z^{4}|HVVVV\rangle\right) \\ +|VHVHV\rangle+|HVHVV\rangle+z\left(|HHVVV\rangle+|VVHVH\rangle\right)+z^{2}\left(|HVVHV\rangle+|VHVVH\rangle\right) \\ +z^{3}\left(|HVVVH\rangle+|VVHHV\rangle\right)+z^{4}\left(|VHHVV\rangle+|VVVHH\rangle\right) \\ \pm i\left(\begin{matrix} \left(|HVHVH\rangle+|VHVHH\rangle\right)+z\left(|VVHHH\rangle+|HHVHV\rangle\right)+z^{2}\left(|VHHVH\rangle+|HVHHV\rangle\right) \\ +z^{3}\left(|VHHHV\rangle+|HHVVH\rangle\right)+z^{4}\left(|HVVHH\rangle+|HHHVV\rangle\right) \end{matrix}\right) \end{matrix}$$

This state has been approved as optimal by the Mathematica script (see next section), as well as Matlab simulations to check the global rotation invariance, maximum performance and fairness. However, we suppose that this state is not the only state available which satisfies optimal criteria, as different phase combinations may give different results. Further research is needed to confirm or invalidate this.

**Section 4. Mathematica script for optimal state verification**

Below is the Mathematica code used to verify whether a state is invariant under global rotation, as well as fair between users. It takes two inputs, N the number of users / photons, and \[Psi] the normalized *N*-photon state, which has to be expressed in the *N*-photon Hilbert space basis by order of tensorial product of $\left\{|H\rangle,|V\rangle\right\}$. The output corresponds to the probability to measure one *N*-photon state for all base state of the Hilbert space corresponding to a global rotation of angle theta with respect to the $\left\{|H\rangle,|V\rangle\right\}$ basis.

In our case, a state is verified as appropriate if it reaches 3 conditions on the script output:

. the first and last probabilities, respectively corresponding to $\otimes_{N} |H\rangle$ and $\otimes_{N} |V\rangle$ in the rotated basis, should always be 0;

. probabilities should not depend on theta;

. non-zero probabilities should be equal between each other, with their sum equal to 1 (because of the normalization of the state).

In the following script, comments explain the role of each variable or function. In addition to N, user should specify which state to check by entering a vector of coefficients in the tensorial basis. One example from the article is already given for each case N=3, 4 and 5. By copying and pasting this script into Mathematica with no modification into a .nb file and running the corresponding cell, the output should be the probabilities to get HHH, HHV, HVH, HVV, VHH, VHV, VVH, VVV respectively, given the ideal state $|\psi\rangle_{3}$ from the main article and a rotation by a random angle theta.

(*Situation with N photons, first 2 lines to reinitialize between 2 runs*)

Unprotect[R,r,Rtemp1,Rtemp2,i,j,theta,N,\[Psi],\[Rho],rotate\[Rho],base,Ndim,EvaluateBase,EvaluateVector];

Clear[R,r,Rtemp1,Rtemp2,i,j,theta,N,\[Psi],\[Rho],rotate\[Rho],base,Ndim,EvaluateBase,EvaluateVector];

N=3;(*Number of photons*)

r={{Cos[theta],-Sin[theta]},{Sin[theta],Cos[theta]}};(*Elementary rotation matrix for|H>,|V>basis*)

base={};For[i=1,i<=2^N,i++,base=Union[{UnitVector[2^N,i]},base]];(*Base vectors of the N-photon Hilbert space*)

R=Array[0&,{2^N,2^N}];(*Initialization of the generalized rotation matrix*)

Ndim=1;Rtemp1=r;

While[Ndim<N,{Rtemp2=Array[0&,{2^(Ndim+1),2^(Ndim+1)}];For[i=0,i<2^(Ndim+1),i++,For[j=0,j<2^(Ndim+1),j++,Rtemp2[[i+1,j+1]]=Rtemp1[[Floor[i/2]+1,Floor[j/2]+1]] r[[Mod[i,2]+1,Mod[j,2]+1]]]],Rtemp1=Rtemp2,Ndim++}]

R=FullSimplify[Rtemp2,theta\[Element]Reals];(*Rotation matrix used*)

\[Rho][\[Psi]_]:=TensorProduct[\[Psi],Conjugate[\[Psi]]];(*Density matrix of input state*)

rotate\[Rho][\[Psi]_]:=R.\[Rho][\[Psi]].ConjugateTranspose[R];(*Rotation of the density matrix*)

EvaluateVector[i_,\[Psi]_]:=Abs[Conjugate[{base[[i]]}].rotate\[Rho][\[Psi]].base[[i]]];(*Evaluate the probability along each vector*)

EvaluateBase[\[Psi]_]:=Array[EvaluateVector[#,\[Psi]]&,2^N];(*Evaluation of probabilities vs theta*)

(*3-photon state invariant under global polarization basis rotation,fair for the players and with minimum conflict*)z=Exp[2I \[Pi]/3];

(*Base: |HHH>,|HHV>,|HVH>,|HVV>,|VHH>,|VHV>,|VVH>,|VVV>*)

\[Psi]=1/Sqrt[6] {0,1,z,I z^2,z^2,I z,I,0};

(*4-photon states invariant under global polarization basis rotation,fair for the players and with minimum conflict*)

(*Base: |HHHH>,|HHHV>,|HHVH>,|HHVV>,|HVHH>,|HVHV>,|HVVH>,|HVVV>,|VHHH>,|VHHV>,|VHVH>,|VHVV>,|VVHH>,|VVHV>,|VVVH>,|VVVV>*)

(*z=Exp[2\[ImaginaryI] \[Pi]/3];

\[Psi]=1/Sqrt[8] {0,1,\[ImaginaryI],0,-1,0,0,\[ImaginaryI],-\[ImaginaryI],0,0,1,0,-\[ImaginaryI],-1,0};

(*\[Psi]=1/Sqrt[6] {0,0,0,1,0,z,z^2,0,0,z^2,z,0,1,0,0,0};*)*)

(*5-photon state invariant under global polarization basis rotation,fair for the players and with minimum conflict*)

(*Base: |HHHHH>,|HHHHV>,|HHHVH>,|HHHVV>,|HHVHH>,|HHVHV>,|HHVVH>,|HHVVV>,|HVHHH>,|HVHHV>,|HVHVH>,|HVHVV>,|HVVHH>,|HVVHV>,|HVVVH>,|HVVVV>,|VHHHH>,|VHHHV>,|VHHVH>,|VHHVV>,|VHVHH>,|VHVHV>,|VHVVH>,|VHVVV>,|VVHHH>,|VVHHV>,|VVHVH>,|VVHVV>,|VVVHH>,|VVVHV>,|VVVVH>,|VVVVV>*)

(*z=Exp[2\[ImaginaryI] \[Pi]/5];

\[Psi]=Array[0&,2^5];

(*4/1 and 1/4 terms*){\[Psi][[2]],\[Psi][[3]],\[Psi][[5]],\[Psi][[9]],\[Psi][[17]],\[Psi][[16]],\[Psi][[24]],\[Psi][[28]],\[Psi][[30]],\[Psi][[31]]}=1/Sqrt[30]{1,z,z^2,z^3,z^4,\[ImaginaryI] z^4,\[ImaginaryI] z^3,\[ImaginaryI] z^2,\[ImaginaryI] z,\[ImaginaryI]};

(*3/2 and 2/3 terms*){\[Psi][[4]],\[Psi][[6]],\[Psi][[7]],\[Psi][[10]],\[Psi][[11]],\[Psi][[13]],\[Psi][[18]],\[Psi][[19]],\[Psi][[21]],\[Psi][[25]],\[Psi][[8]],\[Psi][[12]],\[Psi][[14]],\[Psi][[15]],\[Psi][[20]],\[Psi][[22]],\[Psi][[23]],\[Psi][[26]],\[Psi][[27]],\[Psi][[29]]}=1/Sqrt[30]{\[ImaginaryI] z^4,\[ImaginaryI] z,\[ImaginaryI] z^3,\[ImaginaryI] z^2,\[ImaginaryI],\[ImaginaryI] z^4,\[ImaginaryI] z^3,\[ImaginaryI] z^2,\[ImaginaryI],\[ImaginaryI] z,z,1,z^2,z^3,z^4,1,z^2,z^3,z,z^4};*)

FullSimplify[EvaluateBase[\[Psi]],theta\[Element]Reals]
